# Innate Immune Pathways in Host Defense

**DOI:** 10.1155/2012/708972

**Published:** 2012-12-30

**Authors:** Thirumala-Devi Kanneganti, Mohamed Lamkanfi, Amal O. Amer

**Affiliations:** ^1^Department of Immunology, St. Jude Children's Research Hospital, MS 351, Room E7004, 262 Danny Thomas Place, Memphis,TN 38105-3678, USA; ^2^Department of Biochemistry, Ghent University, 9000 Gent, Belgium; ^3^VIB Department of Medical Protein Research, 9000 Ghent, Albert Baertsoenkaai 3, 9000 Ghent, Belgium; ^4^Department of Internal Medicine, The Ohio State University, Columbus, OH 43210, USA

The innate immune system is a critical component of host defense against invading microbial pathogens. It is responsible for mounting proper inflammatory and repair responses that contribute to the elimination of the invading pathogen and for instructing the adaptive immune system to develop a prolonged immunity against microbial pathogens. This is accomplished through the regulation of transcriptional and posttranslational programs that culminate in the production of proinflammatory cytokines and chemokines, the induction of type I and II interferon responses and autophagy responses, and the induction of programmed cell death modes that eliminate infected host cells and expose intracellular pathogens to surveillance by the immune system. This issue includes eight published papers which are discussing the following issues. 

In the article “*The EGF receptor and HER2 participate in TNF*-**α**-*dependent MAPK activation and IL-8 secretion in intestinal epithelial cells*,” by H. B. Jijon et al., the authors provide evidence that TNF activates one or more metalloproteinases leading to the release of TGF-*α* in intestinal epithelial cells.

In the article “*Innate immune cells in liver inflammation*,” by E. Liaskou et al., the authors discuss the innate immune cells that take part in human liver inflammation, and their roles in both resolution of inflammation and tissue repair. 

In the article “*Optimizing dendritic cell-based immunotherapy*: *Tackling the complexity of different arms of the immune system*,” by I. Brussel et al., the authors explore the molecular and cellular mechanisms underlying adequate immune responses and focus on most favourable DC culture regimens and activation stimuli in humans. Also, they envisage that by combining each of the features outlined in the current paper into a unified strategy, DC-based vaccines may advance to a higher level of effectiveness.

In the article “*Danger signals activating the immune response after trauma*,” by S. Hirsiger et al., the authors focuse on the role of the dual function DAMPs in the initiation of the immune response after trauma. Moreover, they shed light on recently discovered mechanisms of activation of innate immunity by mitochondrial DAMPs released from disrupted cells which bear bacterial molecular motifs similar to PAMPs due to their endosymbiotic origin.

In the article “*Guilty molecules*, *guilty minds? The conflicting roles of the innate immune response to traumatic brain injury*” by S. Hellewell and M. Morganti-Kossmann, the authors discuss the positive, negative, and often conflicting roles of the innate immune response to TBI in both an experimental and clinical settings and highlights recent advances in the search for therapeutic candidates for the treatment of TBI.

In the article “*Interplay between human cytomegalovirus and intrinsic/innate host responses*: *A complex bidirectional relationship*” by G. Rossini., the authors review the viral and cellular partners that mediate early host responses to HCMV with regard to the interaction between structural components of virions (viral glycoproteins) and cellular receptors (attachment/entry receptors, toll-like receptors, and other nucleic acid sensors) or intrinsic factors (PML, hDaxx, Sp100, viperin, interferon inducible protein 16), the reactions of innate immune cells (antigen presenting cells and natural killer cells), the numerous mechanisms of viral immunoevasion, and the potential exploitation of events that are associated with early phases of virus-host interplay as a therapeutic strategy.

In the article “*Lipopolysaccharides: From Erinyes to Charites*” by A. Focà et al., the authors focuse on recent data supporting a beneficial activity of both typical and atypical endotoxins. Such novel perspective looks promising for the development of new drugs for the prevention and therapy of several human diseases.

In the article “*Essential role of mast cells in the visceral hyperalgesia induced by T*. *spiralis infection and stress in rats*” by C.-Q. Yang et al., the authors show that the visceral hyperalgesia cannot be triggered by stress in MCs-deficient rats, although both stress and infection play an important role in visceral hyperalgesia in wild control rats.

*Thirumala-Devi Kanneganti* *Thirumala-Devi Kanneganti* *Mohamed Lamkanfi* *Mohamed Lamkanfi* *Amal O. Amer* *Amal O. Amer*

